# Loss of chaperone-mediated autophagy is associated with low vertebral cancellous bone mass

**DOI:** 10.1038/s41598-022-07157-9

**Published:** 2022-02-24

**Authors:** Nisreen Akel, Ryan S. MacLeod, Stuart B. Berryhill, Dominique J. Laster, Milena Dimori, Julie A. Crawford, Qiang Fu, Melda Onal

**Affiliations:** 1 Department of Physiology and Cell Biology, University of Arkansas for Medical Sciences, Little Rock, AR USA; 2 Center for Musculoskeletal Disease Research, University of Arkansas for Medical Sciences, Little Rock, AR USA; 3 Division of Endocrinology, University of Arkansas for Medical Sciences, Little Rock, AR USA; 4 Genetic Models Core, University of Arkansas for Medical Sciences, Little Rock, AR USA; 5 Bone Biomechanics, Histology and Imaging Core (BHIC), University of Arkansas for Medical Sciences, Little Rock, AR USA

**Keywords:** Autophagy, Bone

## Abstract

Chaperone-mediated autophagy (CMA) is a protein degradation pathway that eliminates soluble cytoplasmic proteins that are damaged, incorrectly folded, or targeted for selective proteome remodeling. However, the role of CMA in skeletal homeostasis under physiological and pathophysiological conditions is unknown. To address the role of CMA for skeletal homeostasis, we deleted an essential component of the CMA process, namely *Lamp2a,* from the mouse genome. CRISPR-Cas9-based genome editing led to the deletion of both *Lamp2a* and *Lamp2c,* another *Lamp2* isoform, producing Lamp2AC global knockout (L2ACgKO) mice. At 5 weeks of age female L2ACgKO mice had lower vertebral cancellous bone mass compared to wild-type (WT) controls, whereas there was no difference between genotypes in male mice at this age. The low bone mass of L2ACgKO mice was associated with elevated RANKL expression and the osteoclast marker genes *Trap* and *Cathepsin K*. At 18 weeks of age, both male and female L2ACgKO mice had lower vertebral cancellous bone mass compared to WT controls. The low bone mass of L2ACgKO mice was associated with increased osteoclastogenesis and decreased mineral deposition in cultured cells. Consistent with these findings, specific knockdown of *Lamp2a* in an osteoblastic cell line increased RANKL expression and decreased mineral deposition. Moreover, similar to what has been observed in other cell types, macroautophagy and proteasomal degradation were upregulated in CMA-deficient osteoblasts in culture. Thus, an increase in other protein degradation pathways may partially compensate for the loss of CMA in osteoblasts. Taken together, our results suggest that CMA plays a role in vertebral cancellous bone mass accrual in young adult mice and that this may be due to an inhibitory role of CMA on osteoclastogenesis or a positive role of CMA in osteoblast formation or function.

## Introduction

In all cell types, stability and renewal of the proteome are maintained by fine-tuned control of protein degradation pathways. Dysfunction and decline in protein clearance mechanisms have been suggested to contribute to compromised cellular function in lysosomal storage diseases and multiple age-related disorders ^1–4^. One of the protein degradation mechanisms implicated in these diseases is autophagy. Autophagy is a proteostasis pathway in which select cellular components are directed to lysosomes for degradation^5^. Autophagy is separated into three types based on the delivery mechanisms utilized to transfer the targets to lysosomes: macroautophagy, chaperone-mediated autophagy, and microautophagy^3^. In macroautophagy, cellular targets are encapsulated by a double membrane vesicle termed an autophagosome and delivered to lysosomes for degradation. Previous studies have shown that macroautophagy in osteoblast-lineage cells contributes to skeletal homeostasis^6–10^. Specifically, we and others have shown that deletion of *Atg7*, which is essential for macroautophagy, in osteoblasts and osteocytes causes low bone mass and fractures^6,7,10^. However, whether other types of autophagy contribute to bone homeostasis is unknown.

**Figure 1:**
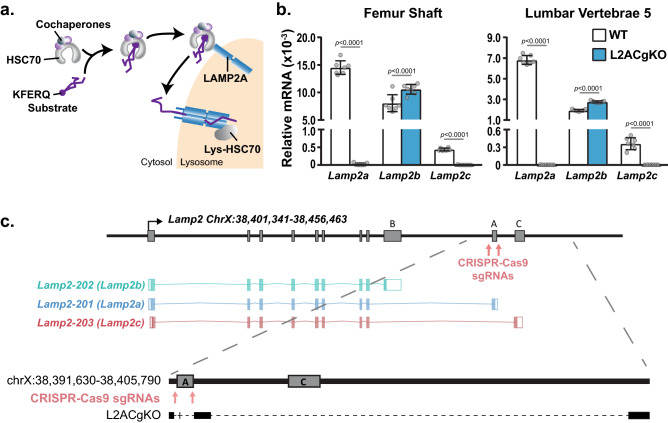
Production of LAMP2AC global knockout mice. **(a)** Illustration of chaperone-mediated autophagy (CMA). HSC70, Heat shock cognate 71 kDa protein; Lys-HSC70, lysosomal HSC70; KFERQ, pentapeptide CMA recognition motif. (**b)** Femur shafts and lumbar vertebra 5 were isolated from 18-week-old male wild type (WT) and LAMP2AC global knockout (L2ACgKO) mice. mRNA levels of *Lamp2* isoforms in these tissues were measured by quantitative real-time PCR (qRT-PCR) and normalized to β-actin. Bars indicate mean ± standard deviation (STDEV), n = 7 mice per genotype. mRNA values of each isoform from WT and L2ACgKO groups were compared by Welch's *t*-test. **c**, Illustration of Chromosome X (Chr X): 38,401,341 -38,456,463 containing the *Lamp2* gene. Schematic of spliced mRNAs encoding the three *Lamp2* isoforms as designated by Ensembl genome browser (Lamp2 ENSMUSG00000016534, https://useast.ensembl.org/Mus_musculus/Gene/Summary?db=core;g=ENSMUSG00000016534;r=X:37490234-37545331). Red arrows indicate the locations that the CRISPR-Cas9 sgRNAs target. Large dashed lines lead to the expanded view of the ChrX:38,391,630-38,405,790 region in WT mice and the sequencing result of the same region in L2ACgKO mice. Black bars represent DNA, gray boxes indicate exons, and small dashed lines indicate deleted/missing regions.

Unlike macroautophagy, chaperone-mediated autophagy (CMA) does not utilize autophagosomes. Instead, selective protein cargo containing a specific pentapeptide sequence (KFERQ-like sequence) is recognized and transported to the lysosomal surface by a chaperone complex (Fig. 1a)^3^. Once at the lysosomal surface, the target proteins are unfolded and transferred into the lysosome one by one via a translocation system that uses lysosome-associated membrane protein type 2A (LAMP2A)^4^. Due to the differences between transport mechanisms, macroautophagy can degrade protein aggregates, organelles, and bulk contents of cytosol, while CMA can only degrade proteins that contain a KFERQ-like recognition motif. While this limits potential targets for CMA, it is important to note that approximately 30% to 40% of proteins in the mammalian proteome display canonical KFERQ-like motifs^11^. Moreover, post-translational modifications such as phosphorylation or acetylation can also produce KFERQ-like targeting motifs, thereby expanding the number of potential protein targets for CMA^3,11^.

Like macroautophagy, CMA degrades excess or damaged proteins in response to stressors such as starvation^12^ or oxidative^13,14^, hypoxic^15^, or genotoxic stress^16^. In line with the role of CMA in responding to these stressors, mice lacking CMA in hepatocytes accumulate oxidized proteins and aggregates prematurely as they age^17^. In addition to the stress response, clearance of proteins by CMA as part of proteome remodeling has been shown to contribute to a wide array of physiological^3,18,19^ and pathophysiological^3,20–23^ processes. For example, CMA has been shown to clear key glycolytic and lipogenesis enzymes, and thereby contribute to glucose and lipid metabolism in hepatocytes^24^, embryonic stem cells^25^, macrophages^26^, and hematopoietic stem cells (HSC)^27^. In addition to its role in metabolic regulation^19,28^, CMA has also been shown to contribute to transcriptional regulation^29^, the immune response^18^, and cell cycle control^16^.

To examine the role of CMA in bone, we created mice that lack CMA and analyzed the impact on the skeleton at 5 and 18 weeks of age. Our results demonstrate that CMA contributes to cancellous bone mass accrual in young mice.

## Results

### Production of CMA deficient mice

To inactivate CMA in mouse cells, we utilized the CRISPR-Cas9 system to delete the *Lamp2a*-specific exon (*Lamp2 Exon 9A*) from the mouse genome (Fig. 1c). LAMP2A is one of the three isoforms encoded by the *Lamp2* gene^30^. These isoforms, namely LAMP2A, LAMP2B, and LAMP2C, differ in their transmembrane and cytosolic domains^31–33^. Amongst these three isoforms, only LAMP2A is necessary for CMA as it has the unique cytosolic domain required to interact with the substrate-chaperone complex^34^. In addition, because the LAMP2A multimeric complex is essential for the translocation of CMA substrates into the lysosome, LAMP2A levels are the essential, rate-limiting component of the CMA process^24,27,35^. While the expression level of the three *Lamp2* isoforms varies in different tissues (Table 1), *Lamp2c* is expressed at very low levels in bone, kidney, and liver (Fig. 1b and S. Fig. 1a–f). This is in line with previous studies that suggest *Lamp2c* expression is restricted to certain tissues and cell types, such as brain, heart, skeletal muscle, B cells and neurons^36,37^. LAMP2B, which has been proposed to facilitate autophagosome and lysosome fusion and thereby participate in macroautophagy, is expressed at comparable levels to LAMP2A in bone (Fig. 1b and S. Fig. 1a–f).

**Table 1:** Differential expression of *Lamp2* isoforms in different tissues.

Ct (log2)	Lumbar vertebrae		Femur shaft		Kidney		Liver	
	Female	Male	Female	Male	Female	Male	Female	Male
*Lamp2a*	23.5 ± 0.4	24.8 ± 0.2	27.0 ± 0.7	26.0 ± 0.6	24.1 ± 0.1	21.7 ± 0.3	21.7 ± 0.2	21.2 ± 0.3
*Lamp2b*	25.9 ± 0.4	26.6 ± 0.3	28.8 ± 0.6	27.1 ± 0.8	24.6 ± 0.2	24.8 ± 0.3	24.6 ± 0.2	25.0 ± 0.1
*Lamp2c*	29.6 ± 0.6	29.1 ± 0.3	33.3 ± 0.9	32.0 ± 1.7	26.2 ± 0.3	26.9 ± 0.4	28.5 ± 0.3	31.6 ± 0.2
*β-actin*	16.2 ± 0.5	17.6 ± 0.3	21.5 ± 0.8	19.9 ± 0.7	17.9 ± 0.2	17.8 ± 0.3	21.3 ± 0.4	18.8 ± 0.3

Tissues were isolated from the 18-week-old wild type mice. mRNA levels of *Lamp2* isoforms and control housekeeping gene beta-actin (β-actin) were measured by qRT-PCR and indicated as Ct values (log2).

n = 6–10 mice/genotype.

**Figure 2:**
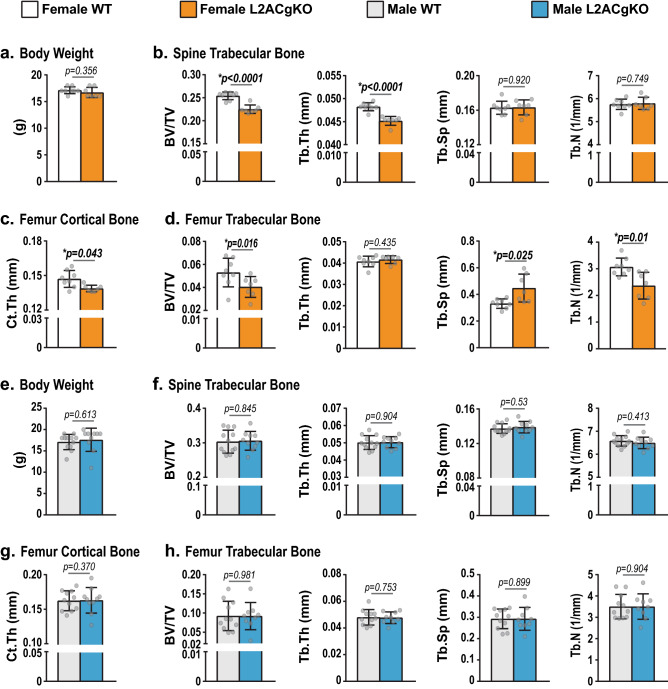
Skeletal analysis of growing L2ACgKO mice. **(a–h)** Body weight measurements (**a,e**) and μCt analysis was performed on bones of 5-week-old female (**a–d**) and male (**e–h**) Lamp2AC global knock-out (L2ACgKO) mice and their wild-type (WT) littermates. (**b,f**) Cancellous bone mass and architecture were analyzed as bone volume over tissue volume (BV/TV), trabecular thickness (Tb.Th), trabecular spacing (Tb.Sp), trabecular number (Tb.N) in lumbar vertebrae 4. (**d,h)** Femoral cancellous bone mass and architecture were analyzed in the distal femur. (**c,g**) Femoral cortical thickness (Ct.Th) was measured at the midshaft. n = 7–12 mice/group. Bars indicate mean ± STDEV. *p* values were calculated and evaluated by Welch's *t*-test; *, p < 0.05.

To delete *Lamp2* exon 9A*,* single guide RNAs (sgRNAs) were designed to direct Cas9 to sites flanking this exon. However, likely due to the highly repetitive nature of this locus, CRISPR-Cas9-based genome editing led to the deletion of both exon 9A and exon 9C (Fig. 1c and S. Fig. 2). As a result, the resulting mice, designated Lamp2AC global knockout (L2ACgKO) mice, lack both *Lamp2a* and *Lamp2c* transcripts in all tissues examined (Fig. 1b and S. Fig. 1). Like previous studies in which *Lamp2a* was deleted specifically from hepatocytes^24^, deletion of *Lamp2a* led to an increase in expression of *Lamp2b* (Fig. 1b and S. Fig. 1).

### Young L2ACgKO female mice have low bone mass

To address whether CMA plays a role during skeletal growth, we assessed the skeletal phenotypes of male and female mice at 5 weeks of age. At this age, L2ACgKO mice were similar in size to their wild-type (WT) littermates (Fig. 2a,e). MicroCT (µCT) analysis of 5-week-old CMA-deficient female mice showed that they had lower vertebral cancellous bone volume compared to WT controls (Fig. 2b). This decrease in vertebral cancellous bone was associated with a decrease in trabecular thickness (Fig. 2b). Similarly, femoral cancellous bone volume was lower in CMA-deficient female mice compared to the controls (Fig. 2d). The decrease in femoral cancellous bone was associated with an increase in trabecular separation and a decrease in trabecular number (Fig. 2d). μCT analysis at the femoral midshaft revealed that female L2ACgKO mice also had a lower cortical thickness compared to controls (Fig. 2c). Unlike the female L2ACgKO mice, bone mass and architecture of male CMA-deficient mice were similar to their WT littermates at this age (Fig. 2f–h).

**Figure 3:**
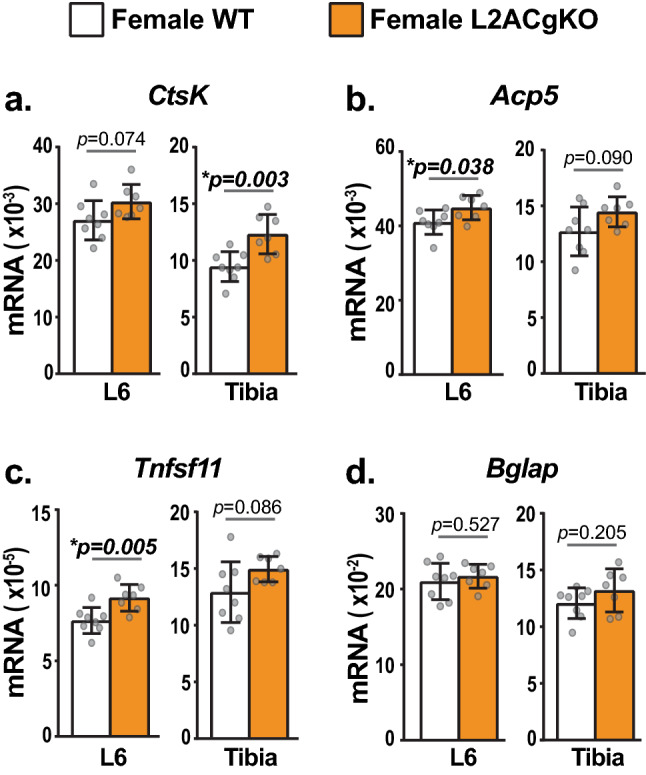
Gene expression analysis of bone formation and resorption marker genes in L2ACgKO bones. qRT-PCR was used to measure mRNA levels of target genes in lumbar vertebrae 6 (L6), and tibias of 5-week-old female Lamp2AC global knock-out (L2ACgKO) mice and their age-matched controls (wild-type mice, WT). (**a–d**) mRNA levels of osteoclast marker genes C*athepsin K (CtsK)* and *Trap (Acp5)* (**a,b**)*; Tnfsf11 encoding* pro-resorptive cytokine RANKL (**c**), and osteoblast marker gene *Bglap* encoding Osteocalcin (**d**) were measured. Levels of target genes were normalized to the housekeeping gene β-actin. n = 7–8 mice/group. Bars indicate mean ± STDEV. *p* values were calculated and evaluated by Welch's *t*-test.; *, p < 0.05.

We next measured mRNA levels of genes commonly used as markers for osteoblasts and osteoclasts. Expression of osteoclast marker genes *CtsK* and *Acp5,* encoding Cathepsin K and Tartrate-resistant acid phosphatase (TRAP), respectively, were slightly elevated in bones of female L2ACgKO mice compared to WT controls (Fig. 3a,b). This elevation in osteoclast marker genes was associated with an increase in the mRNA encoding the osteoclastogenic cytokine *Tnfsf11 (Rankl)* in lumbar vertebra 6 (L6) and a trend towards an increase in the tibia (Fig. 3c). Expression of the osteoblast marker gene *Bglap,* which encodes osteocalcin, was similar in L2ACgKO and WT mice (Fig. 3d). In line with a female-specific phenotype at this age, the expression of osteoblast and osteoclast marker genes were similar between male L2ACgKO mice and their WT littermates (S. Fig. 3). Collectively, these results suggest that the lower bone mass of female L2ACgKO mice may be due to an increase in osteoclasts.

### Adult male and female CMA-deficient mice have low cancellous bone mass

**Figure 4:**
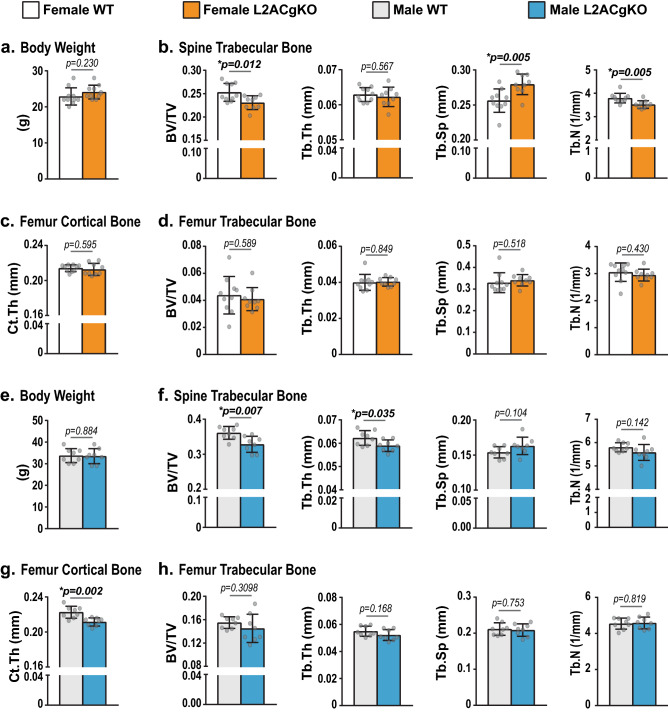
Skeletal analysis of young adult L2ACgKO mice. **(a–h)** Body weight measurements (**a,e**) and μCt analysis was performed on lumbar vertebrae 4 (L4) and femurs of 18-week-old female (**a–d**) and male (**e–h**) Lamp2AC global knock-out (L2ACgKO) mice and their wild-type (WT) littermates. (**b,f**) Cancellous bone mass and architecture were analyzed as bone volume over tissue volume (BV/TV), trabecular thickness (Tb.Th), trabecular spacing (Tb.Sp), trabecular number (Tb.N) in lumbar vertebrae 4. (**d,h)** Femoral cancellous bone mass and architecture were analyzed in the distal femur. (**c,g**) Femoral cortical thickness (Ct.Th) was measured at the midshaft. n = 8–10 mice/group. Bars indicate mean ± STDEV. *p* values were calculated and evaluated by Welch's *t*-test; *, p < 0.05.

To address the role of CMA in young adult mice, we assessed the skeletal phenotype of male and female mice at 18 weeks of age. Eighteen-week-old L2ACgKO mice were similar in size to their WT littermates (Fig. 4a,e). At this age, both male and female L2ACgKO mice had lower vertebral cancellous bone volume compared to WT controls (Fig. 4b,f). In female L2ACgKO mice, the decline in bone volume was associated with a decline in trabecular number and an increase in trabecular separation (Fig. 4b). In male L2ACgKO mice, the decline in cancellous bone volume was associated with a decline in trabecular thickness (Fig. 4f). Significant changes in cancellous bone mass and architecture were not observed in the femurs of male or female L2ACgKO mice (Fig. 4d,h). Male, but not female, L2ACgKO mice had lower femoral cortical thickness compared to WT controls (Fig. 4c,g). However, the observed decline in cancellous and cortical bone mass of male L2ACgKO mice did not alter the strength properties of their femurs or vertebra (S. Fig. 4).

**Figure 5:**
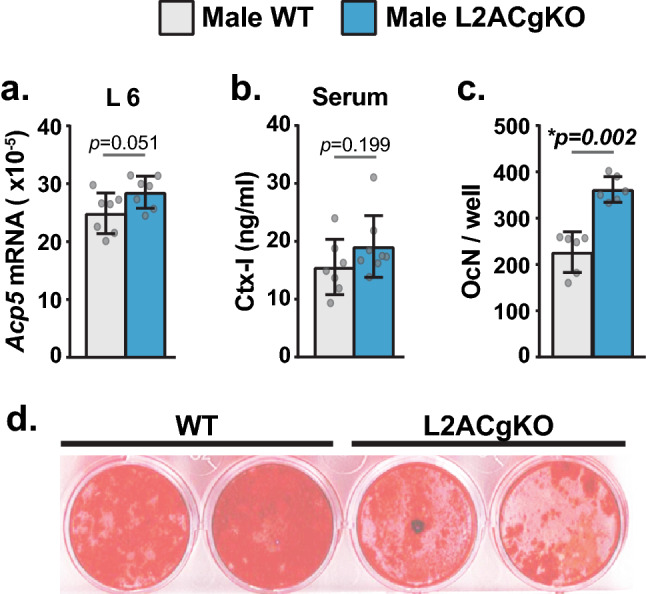
CMA-deficiency is associated with increased osteoclastogenesis. **(a,b)** Bone resorption markers were measured in 18-week-old male Lamp2AC global knock-out (L2ACgKO) mice and their control littermates (wild type mice, WT). (**a)** qRT-PCR analysis was used to measure mRNA levels of osteoclast marker gene TRAP (*Acp5*) in lumbar vertebrae 6 (L6). TRAP mRNA levels were measured and normalized to the housekeeping gene β-actin. (**b**) Circulating bone resorption marker C-terminal telopeptide of type I collagen (Ctx-I) was measured by ELISA. (**a,b**) n = 7–8 mice/group. Bars indicate mean ± STDEV. *p* values were calculated and evaluated by student’s *t*-test; *, p < 0.05. (**c**) Bone marrow was isolated from 4-month-old L2ACgKO and WT mice. Bone marrow of 3 mice/genotype were pooled and plated for in vitro osteoclast formation assays. Osteoclast number per well (OcN/well) was quantified using 6 wells/genotype. Bars indicate mean ± STDEV. *p* values were calculated and evaluated by Welch's *t*-test; *, p < 0.05. Osteoclast formation assay was performed two additional times with bone marrow isolated from different sets of mice. These data and representative images of the osteoclast cultures can be found in S. Fig. 5d, Bone marrow was isolated from 4-month-old L2ACgKO and WT mice. Cells were differentiated into osteoblasts by culturing with osteogenic media for 28 days. Alizarin Red staining was used to determine mineral deposition by differentiated osteoblasts.

To assess the cellular basis underlying the low cancellous bone mass of L2ACgKO mice, we measured osteoclast and osteoblast marker genes, as well as serum markers of bone formation and resorption. At 18-weeks-of-age, all of these parameters were similar in female L2ACgKO mice and their controls (data not shown). In 18-week-old male L2ACgKO mice, *Acp5* expression and serum C-terminal telopeptide of type I collagen (CTX-I) levels trended higher compared to their WT littermates (Fig. 5a,b). Moreover, osteoclast formation was higher in cultures of bone marrow cells isolated from L2ACgKO mice compared to those from WT mice (Fig. 5c and S. Fig. 5). Taken, together these results suggest that the low cancellous bone mass of male L2ACgKO mice may be due to increased osteoclastogenesis. Osteoblast marker gene expression and serum bone formation markers were similar in male L2ACgKO mice and their WT littermates (data shown). However, L2ACgKO osteoblasts exhibited reduced mineral deposition in culture (Fig. 5d). These results suggest that reduced osteoblast proliferation, formation or activity, although not to levels that would be reflected in gene expression or circulating markers, may also be contributing to the low bone mass of L2ACgKO mice.

### *Lamp2a* suppression in osteoblasts increases RANKL expression and decreases mineral deposition

**Figure 6:**
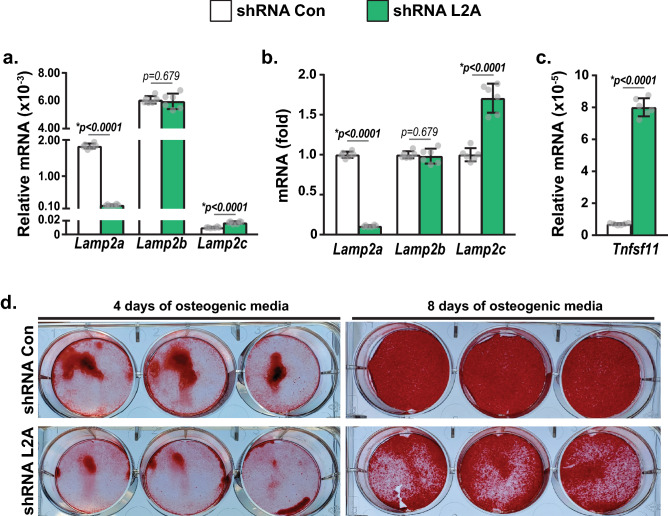
CMA deficiency increases RANKL expression and decreases mineral deposition of an osteoblastic cell line. *Lamp2a* was knocked down in osteoblastic UAMS-32 cells and plated into 6 well plates for gene expression or mineralization assays. (**a–c**) qRT-PCR analysis of three *Lamp2* isoforms and *Tnfsfs11 (Rankl)* in osteoblastic UAMS-32 cells that stably express scrambled shRNA (shRNA Con) or shRNA targeting LAMP2A (shRNA L2A). mRNA levels of all transcripts were normalized to the housekeeping gene β-actin. n = 6 wells/group. Bars indicate mean ± STDEV. *p* values were calculated and evaluated by Welch's *t*-test; *, p < 0.05. Knockdown of *Lamp2a* and the following gene expression was performed an additional time independently and shown in S. Fig. 6a–c. (**d**) shRNA Con and shRNA L2A cells were cultured in osteogenic media. Mineral deposition was measured by Alizarin Red staining on day 4 and day 8 of culture.

We next wanted to determine if deletion of *Lamp2a*, but not *Lamp2c*, increased the ability of osteoblasts to support osteoclastogenesis and reduced their mineral deposition. For this purpose, we knocked down *Lamp2a* expression in UAMS-32 osteoblastic cell line and calvarial osteoblasts isolated from wild-type mice. Similar to what is observed in bone shafts enriched in bone cells, *Lamp2c* expression was very low compared to the other two *Lamp2* isoforms in both UAMS-32 cells (Fig. 6a and S. Fig. 6a) and calvarial osteoblasts (S. Fig. 6d). *Lamp2a* knockdown caused elevation of *Lamp2c* levels in UAMS-32 cells, but not in calvarial osteoblasts (Fig. 6a,b, S. Fig. 6a,b,d,e). However, knockdown of *Lamp2a* in either type of cultured osteoblasts increased RANKL expression (Fig. 6c, S. Fig. 6c,f). These data suggest lack of *Lamp2a*, and therefore lack of CMA, causes an increase in RANKL expression, and that this observed increase in RANKL is independent of changes in *Lamp2c* levels. This is consistent with the increase in RANKL expression observed in growing L2ACgKO mice (Fig. 3c). As bone resorption trended towards an increase in young adult mice (Fig. 5a–c), it is likely that RANKL also increased in CMA-deficient osteoblasts of young adult mice. However, such increases could not be detected in whole bone preparations (data not shown), perhaps because osteoblasts are one of many cell types in bone that produce RANKL. Similar to cultured L2ACgKO osteoblasts, CMA-deficient UAMS-32 cells cultured in osteogenic media for 4 or 8 days exhibited reduced mineral deposition compared to controls (Fig. 6d). These results suggest that CMA may play a role in the differentiation of bone-forming osteoblasts or their activity. Taken together, these data suggest that lack of *Lamp2a*, but not *Lamp2c*, underlie the changes observed in L2ACgKO osteoblasts.

### Other protein degradation pathways are upregulated in CMA-deficient osteoblasts

The elimination of CMA from fibroblasts and hepatocytes has been shown to cause a compensatory increase in macroautophagy and the ubiquitin–proteasome system^17,35^. Moreover, these compensatory increases in other protein degradation pathways have been shown to maintain proteostasis in livers of young mice^24^. To address whether a similar compensation may be taking place in CMA-deficient osteoblasts, we isolated calvarial osteoblasts from WT and L2ACgKO mice and quantified the activity of macroautophagy and the ubiquitin–proteasome system.

**Figure 7:**
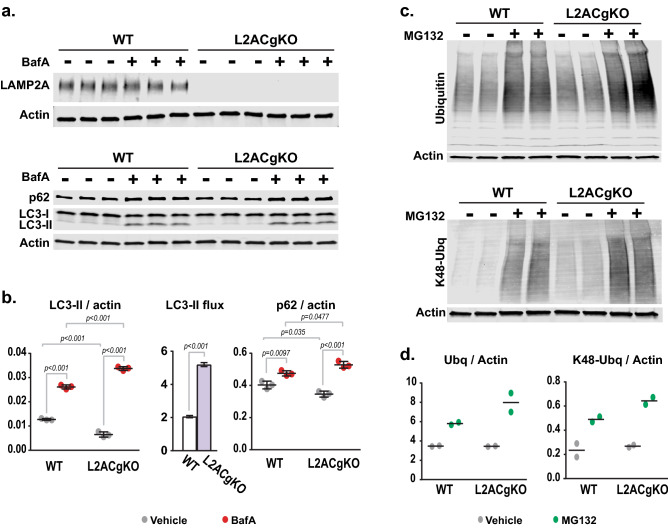
Macroautophagy and proteasomal degradation increase in the absence of CMA. Calvarial osteoblasts were isolated from Lamp2AC global knock-out (L2ACgKO) mice and their control littermates (wild-type mice, WT). (**a**) Calvarial osteoblasts isolated from one mouse per genotype were plated in 6 well plates and cultured with vehicle (DMSO) or 100 μM Bafilomycin A (BafA) for 4 h. LAMP2A, LC3, and p62 protein levels were determined by western blot analysis. (**b**) LC3-II and p62 levels were quantified and normalized to actin levels. For LC3-II flux analysis BafA treatment of each genotype was normalized its vehicle treatment. n = 3 wells/treatment per genotype. Lines indicate mean ± STDEV. For comparisons of LC3-II/actin and p62/bactin levels *p* values were calculated and evaluated by Two-way ANOVA. For LC3-II flux comparison *p* value was calculated and evaluated by student’s *t*-test; *, p < 0.05. This experiment was repeated using calvarial cells isolated from another set of WT and L2ACgKO mice. These results can be found in S. Fig. 7c. **(c,d**) Calvarial osteoblasts isolated from one mouse per genotype were plated in 6 well plates and cultured with vehicle (DMSO) or 50 μM of proteasome inhibitor MG132 for 6 h. The levels of ubiquitinated proteins and K48-linked ubiquitinated proteins were quantified by Western blot analysis. Full-length blots can be found in S. Fig. 7.

To assess the autophagy levels, we measured the flux of microtubule-associated protein 1A/1B-light chain 3 (LC3) in calvarial osteoblasts. As autophagosomes are formed, the cytoplasmic form of LC3 (LC3-I) is lipidated to LC3-phosphatidylethanolamine conjugate (LC3-II) and incorporated into the autophagosomal membrane. Once autophagosomes fuse with lysosomes, LC3-II is degraded in the lysosome along with the contents of the autophagosome. Based on this, LC3-II flux is commonly used as a measure of autophagic flux. In CMA-deficient calvarial osteoblasts, suppression of autophagosome-lysosome fusion with Bafilomycin A (BafA) revealed that CMA-deficient osteoblasts have higher levels of LC3-II flux compared to WT controls (Fig. 7a,b and S. Fig. 7c), suggesting elevated macroautophagy levels. Next, we measured levels of p62, a classical autophagy substrate. p62 levels were slightly lower in CMA-deficient osteoblasts, and prevention of p62 degradation by macroautophagy led to higher p62 levels in CMA-deficient osteoblasts compared to WTs. These results suggest that the turnover of p62 by macroautophagy is higher in CMA-deficient osteoblasts (Fig. 7a,b and S. Fig. 7c). Taken together, these results suggest that macroautophagy is increased in the absence of CMA.

To assess the activity level of the ubiquitin–proteasome system, we measured the flux of proteasome substrates, namely ubiquitinated proteins, as well as the more specific classical proteasome substrate K48-linked ubiquitinated proteins. Under basal conditions, both ubiquitinated and K48-linked ubiquitinated protein levels were similar between WT and L2ACgKO cells (Fig. 7c,d). However, when cells were treated with a proteasome inhibitor, L2ACgKO cells accumulated higher levels of ubiquitinated and K48-linked ubiquitinated substrates (Fig. 7c,d), suggesting that more of these substrates were being degraded by the proteasome in L2ACgKO cells.

## Discussion

Previous studies in non-osseous cell types have shown that while different types of autophagy crosstalk with each other to maintain cellular proteostasis, they are not redundant. We and others have previously shown that macroautophagy is an essential clearance pathway in the osteoblast lineage^6,7^. However, the functions of other types of autophagy in bone cells are unknown. To address the role of CMA in bone cells, we created a murine model that lacks CMA, namely L2ACgKO mice. Our results showed that global CMA deficiency in young mice caused low vertebral cancellous bone mass in both sexes. However, we noted a sex-based difference in the age at which this phenotype develops. Specifically, female mice exhibited this phenotype at an earlier age. The reasons for this sexual dimorphism are not clear and may be due to the indirect effects of CMA in other tissues. Female L2ACgKO mice had low vertebral bone mass during growth and in adulthood. However, the femoral phenotype of the female L2ACgKO mice was observed during growth, but not in adulthood. In mice, the volume of cancellous bone in the femur is much lower than in vertebral bone. This low volume combined with high variance may have limited our ability to detect differences between genotypes. In addition, unlike vertebral cancellous bone, femoral cancellous bone volume declines rapidly as mice mature, especially in female C57BL/6 mice^38^. Therefore, the lack of a difference in femoral cancellous bone volume may be related to the rapid loss of this bone as mice mature. We also observed lower cortical thickness in 5-week old female and 18-week old male mice that lack CMA. As the change in the cortical thickness was modest and its occurrence was not consistent between sexes and ages, the significance of this finding remains unclear. In contrast, the low vertebral cancellous bone mass in young adult CMA-deficient mice was a consistent finding and thus may reflect an important role for CMA in bone cells.

Similar to what is observed when CMA is eliminated from hepatocytes^24^ or HSCs^27^, the phenotype associated with CMA deficiency in young L2ACgKO mice is rather mild. There are several potential explanations for this. First, CMA elimination may lead to upregulation of other proteostasis pathways. Indeed, our in vitro studies showed that macroautophagy and proteasomal degradation are upregulated in L2ACgKO osteoblasts compared to controls. Similar compensatory mechanisms may also be occurring in osteoclasts. Future studies will be necessary to examine potential crosstalk between macroautophagy and CMA, and to what degree they can compensate for each other in bone cells. Second, as was observed in HSCs^27^, CMA may not be essential for the basal function of osteoblast or osteoclast lineage cells but may be important as a stress response pathway. In line with this, we observed that various cellular stressors, including starvation, oxidative and genotoxic stress, induce CMA in an osteoblastic cell line (data not shown). Future studies will be necessary to determine the functional role of CMA under stress conditions and aging.

We have previously eliminated macroautophagy from osteoblasts and osteocytes by deleting an essential component of the macroautophagy pathway, namely *Atg7*. Unlike the mild phenotype of mice with global CMA deficiency, lack of macroautophagy in osteoblastic cells dramatically lowers bone mass and causes fractures. Moreover, while macroautophagy-deficiency in the osteoblast lineage was associated with low osteoblast and osteoclast numbers, mice with global CMA deficiency exhibit evidence of elevated osteoclast formation. This difference suggests that these two types of autophagy play distinct roles in bone remodeling. However, cell-type-specific loss of function studies will be required to determine if this is the case.

Unexpectedly, we inactivated both LAMP2A and LAMP2C in our model. We and others have shown that in most tissues, *Lamp2c* is the lowest expressed *Lamp2* isoform^36,37^. However, *Lamp2c* expression level varies between tissues and is comparable to other *Lamp2* isoforms in the brain, heart, and skeletal muscle^37^. *Lamp2c* is expressed at the highest level in the brain and this high endogenous *Lamp2c* level has been proposed to mediate lysosomal degradation of RNA in this tissue^36^. Although endogenously expressed at low levels, inflammatory conditions increase *Lamp2c* levels in B cells^37^ and melanoma cells^39^. Strong overexpression of *Lamp2c* in melanoma^39^ and B cells^37^ has been reported to inhibit macroautophagy and CMA in these cell types, suggesting potential roles of LAMP2C in these cell types under inflammatory conditions. However, the role of LAMP2C in bone or other tissues remains unclear. Based on the very low expression of *Lamp2c* in whole bones, bone shafts, calvarial osteoblasts, and osteoblastic cell lines, and our in vitro studies in which specific knockdown of *Lamp2a* in cultured osteoblasts mirrored some of our key findings (such as the elevation of RANKL expression and the decrease in mineral deposition), we attribute the skeletal phenotype of L2ACgKO mice to deletion of *Lamp2a* and therefore lack of CMA. However, we cannot rule out the possibility that lack of *Lamp2c* in bone or other cell types may contribute to the observed phenotype, and future studies that delete only *Lamp2a* in osteoblast lineage cells are necessary to confirm that the low bone mass phenotype we observe is due to the elimination of CMA from this cell type.

Danon disease, also called glycogen storage disease type 2B, is a rare lysosomal storage disorder that is caused by mutations in the *Lamp2* gene. In Danon disease, patients either lack or have very low levels of all three LAMP2 isoforms and therefore have defects in both macroautophagy and CMA^40^. Weakening of the heart muscles leads to cardiomyopathy, causing heart failure and premature death in these patients^41^. Similarly, in a murine model of Danon disease^42^, which lacks all 3 isoforms of LAMP2, half of the LAMP2 null mice died between postnatal days 20 and 40, independent of sex and genetic background. Moreover, all LAMP2 null mice, including the survivors, were smaller than their control littermates. Unlike the LAMP2 null mice, our mice that lack LAMP2A and LAMP2C did not display premature death or decreased body weight. This suggests that the increased premature mortality or reduced size observed in LAMP2 null mice are either due to defects in macroautophagy stemming from the absence of LAMP2B alone or due to the combination of defects in both macroautophagy and CMA. In recent studies, Jansen et al.^43^ have examined the bone mass of LAMP2-deficient male mice at 7 to 14 weeks of age. At this age, they did not observe differences in tibial or calvarial bone mass between LAMP2-deficient mice compared to controls. We cannot make a direct comparison of these results to our own because the LAMP2-deficient mice used by Jansen et al. are on a mixed genetic background, show increased early mortality, and are analyzed at different ages and skeletal sites than our studies.

In summary, we showed that lack of *Lamp2a* and *Lamp2c* contributes to cancellous bone mass accrual in young mice and that this may be due to an inhibitory role of CMA on osteoclastogenesis or a role of CMA in osteoblast formation or function. We also showed that increases in other proteostasis pathways may compensate for the lack of CMA in osteoblasts. Future studies will be necessary to address the extent of crosstalk between CMA and macroautophagy in bone cells, as well as the role of CMA for bone cells under stress conditions such as aging.

## Materials and methods

### Generation of L2ACgKO mice

To target *Lamp2* exon 9A for deletion by CRISPR/Cas9 system, two sgRNAs flanking exon9A were identified and cloned into px330 plasmids. The recognition sequences of these sgRNAs were 5′-AAATCTGTATGCAGGGGGAA-3′ and 5′-AAATTGAAACAGAACACCGG-3′, which were 78 bp upstream from *Lamp2 Exon 9A* and 366 bp downstream of the *Lamp2* stop codon in *Exon 9A*, respectively. pX330 plasmids expressing Cas9 and sgRNAs were injected into the pronuclei of fertilized C57BL/6 J mouse eggs, which were then implanted in the oviducts of pseudopregnant ICR mice. The resultant founders were screened for the deletion of the *Lamp2* exon9A using the following primers: forward 5′-TCATCCCTCCAAAAACCAAG-3′, and reverse 5′-TGAAGGGGAACTGCATAGAA-3′. One founder lacking *Lamp2* Exon9A was identified. Breeding of this founder mouse and all subsequent offspring was performed with C57BL/6 J mice. Genotyping of the offspring was performed with genomic DNA isolated from tail tips and two PCRs, using the following primer sets: one set whose product spans *Lamp2* exon9A forward 5′-GATGGCCCTACGGACTCTCT-3′ and reverse 5′-CCCCCAATGACTGCTTTTTA-3′; and the second set whose product lies within *Lamp2* exon9A forward 5′- AAAGCCAATCTGCATTTTAAGC-3′ and reverse 5′-TCTCAAGCGCCATCATACTG-3′. An additional genotyping PCR for Cas9 was performed in the first generation offspring to ensure that the px330 plasmid was not inserted into the genome. To sequence the *Lamp2* loci after genome editing, the region of interest was amplified using the following primers: forward 5′-GCATTGAGCTCTGTGCTGC-3′ and reverse 5′-TTTTGTTTTTGCTGTCATGGTC-3′. The resulting PCR products were cloned into pCRTM4-TOPO vector according to the TA Cloning^®^ Kit protocol (Invitrogen, cat no. 45-0030) and sequenced using T7 and T3 primers. All mice were provided water and food ad libitum and were maintained on a 12-h light/dark cycle. All animal studies were carried out in accordance with the policies of, and with approval from, the Institutional Animal Care and Use Committee of the University of Arkansas for Medical Sciences. The studies of this manuscript were performed and reported in accordance with the ARRIVE guidelines.

### RNA isolation and gene expression analysis

Dissected murine bone and soft tissues were snap-frozen in liquid nitrogen and stored at –80 °C. Lumbar vertebrae, femur shafts, and livers were homogenized in Trizol Reagent (Life Technologies # 15596018), and RNA was isolated from these tissues per manufacturer’s instructions. Kidneys were homogenized in Trizol Reagent, and the RNA was isolated using a Direct-zol RNA Miniprep Plus Kit (Zymo Research, # R2072) according to the manufacturer’s instructions. RNA concentrations and the 260/280 ratios were determined using a Nanodrop instrument (Thermo Fisher Scientific). 1 µg of RNA from each tissue was used to synthesize cDNA using a High-Capacity cDNA Reverse Transcription Kit (Applied Biosystems #4368814) according to the manufacturer’s instructions. Relative mRNA levels were determined using multiplex quantitative real-time PCR (qRT-PCR) analysis using TaqMan Fast Advanced Master Mix (Applied Biosystems # 4444964), VIC‐labeled *Mouse Actb* (Life Technologies, #4352341E), and FAM‐labeled TaqMan gene expression assays (Life Technologies). The following FAM-labeled assays were used in the gene expression analysis: *Tnfsf11* (Mm00441906_m1), *Tnfrsf11b* (Mm00435454_m1), *Cathepsin K* (Mm00484039_m1), *Acp5* (Mm00475698_m1), *Lamp2a* = *Lamp2-201* (Mm00495274_m1), *Lamp2b* = *Lamp2-202*(Mm00627500_m1), *Lamp2c* = *Lamp2-203* (Mm01317405_m1) and *Bglap* (forward primer 5′-GCTGCGCTCTGTCTCTCTGA-3′, reverse primer 5′-TGCTTGGACATGAAGGCTTTG-3′, FAM labeled probe 5′- FAM-AAGCCCAGCGGCC-NFQ-3′). The relative mRNA levels were determined using the comparative cycle threshold (ΔCt) method^44^.

### Osteoclast assay

Femoral bone marrow samples were flushed using αMEM (Gibco, #11900073) containing 15% Fetal Bovine Serum (Hyclone, # SH3007103) and 1% penicillin–streptomycin-glutamine (Gibco, # 10378016). For each genotype, bone marrow samples from three mice were pooled. The bone marrow samples were homogenized using a syringe with a 22G needle to get a single cell suspension and were then strained through a 70 µm cell strainer. Cells were pelleted at 200 × *g* for 5 min, resuspended in 10 mls of media, and counted. As previously described^45–48^, cells were plated in 24-well plates (6 wells per genotype) at a density of 2 × 10^6^ cells per well in the above media supplemented with β-Glycerolphosphate 10 mM (Sigma, # G9422), L-Ascorbic Acid 50 µM (Sigma # A4544) and 1α,25-dihydroxycholecalciferol 10^–8^ M (Sigma, # D1530). Cells were maintained at 37 °C and 5% CO_2_. Half of the media was changed on day 5. On day 10, cells were fixed with 10% formalin and stained for TRAP. The number of osteoclasts was determined by counting the number of TRAP-positive multinucleated cells (≥ 3nuclei/cell) per well. Osteoclastogenesis assay was performed three independent times (Fig. 5c and S. Fig. 5a).

### Osteoblastic cell culture

Bone marrow cells were harvested from long bones and plated in 12-well plates with a density of 2 × 10^6^ cells per well in osteogenic media (α-MEM containing 15% fetal bovine serum, 1% penicillin/streptomycin/glutamine, 50 μg/ml ascorbic acid, and 10 mM β-glycerolphosphate). Half of the culture medium was changed every 5 days with fresh osteogenic media containing 2 × ascorbic acid (100 μg/ml). After 28 days, the cultures were fixed with 50% ethanol at 4 °C for 15 min, dried and then stained with an aqueous solution of 40 mM alizarin red for 45 min at room temperature.

Osteoblastic UAMS-32 were cultured in α-MEM containing 10% fetal bovine serum, 1% penicillin/streptomycin/glutamine. Calvarial cells (isolated as indicated below) were cultured in alpha MEM containing 20% Fetal Bovine Serum and 1% antibiotics (Penicillin–Streptomycin-Glutamine). To knock down *Lamp2a*, cells were infected with lentivirus expressing shRNA for *Lamp2a* (target sequence GAAGCACTTTGCTCCTTAAGA, GeneCopoeia lentiviral vector psi-LVRU6GP) or scrambled control (GeneCopoeia lentiviral vector psi-LVRU6GP). Cells expressing the shRNAs were selected via puromycin selection. For gene expression analysis cells were plated in 6 well plates, RNA was isolated with Trizol per manufacturer’s instructions and processed for gene expression as indicated in the above section (RNA Isolation and Gene Expression Analysis). For mineral deposition assay, UAMS-32 cells were plated into 6-well plates and cultured in osteogenic media for 4 or 8 days. Next, the cultures were fixed with 50% ethanol, dried and stained with 40 mM alizarin red. Knockdown of *Lamp2a* and the following gene expression, differentiation, and Alizarin Red staining were performed two independent times.

### Serum bone turnover assays

Maxillary blood was collected from each mouse prior to euthanization. Collected blood was maintained at room temperature for a minimum of 30 min, followed by centrifugation at 2000 × *g* for 12 min. The supernatant was transferred into a new tube. Samples were centrifuged again at 2000 × *g* for 12 min. The serum was transferred to a new tube and stored at –20 °C until use. Serum was used to measure bone resorption marker C-terminal telopeptides of collagen I (CTX-I, RatLaps EIA, Immunodiagnostics systems kit, # AC-06F1) and bone formation marker N-terminal propeptide of type I procollagen (PINP, PINP EIA kit, Immunodiagnostics Systems kit, # AC-33F1) according to the manufacturer’s instructions.

### MicroCT analysis

MicroCT analysis was performed on the fourth lumbar vertebrae (L4) and femurs. The femurs and vertebrae were dissected, cleaned of soft tissue, wrapped in saline-soaked gauze, and stored at –20 °C. At the time of scanning, bones were thawed, loaded into a 12.3 mm diameter scanning tube filled with saline. The microCT scans were performed on a model uCT40 (Scanco Biomedical) to generate three-dimensional voxel images (1024 × 1024 pixels) of the bones. Medium resolution scans were obtained (E = 55 kVp, I = 145 uA, integration time = 200 ms). A Gaussian filter (sigma = 0.8, support = 1) was used to reduce noise, and a threshold of 220 was used for all scans. The femur scans spanned from the distal growth plate to the midshaft. The trabecular analysis was performed on the first 151 slices above the growth plate by drawing contours every 20 slices and using voxel counting for bone volume per tissue volume and sphere-filling distance transformation indices. The midshaft cortical measurements were performed by drawing contours to measure the cortical thickness on the first 20 midshaft slices. The whole vertebral body was scanned, and the trabecular analysis was performed by drawing contours every 10 slices on the whole space between the 2 growth plates, excluding the cortical bone. Calibration and quality control of the scanner were performed weekly or monthly as previously described^49^.

### Calvarial osteoblast isolation and culture

Calvariae were dissected from 3- to 5-day-old mice and washed four times with cold PBS. Calvariae were transferred to a 6 well plate and then incubated for 20 min at 37 °C and 5% CO_2_ with digestion media I (alpha MEM, 0.4% Trypsin [Gibco, #25200056], 0.1 mg/mL collagenase P [Sigma, #11213857001] and antibiotics). The plate was shaken vigorously for 20 seconds (s) every 5 minutes (min) during this 20-min incubation time. The released cells were discarded, and the incubation with digestion media was repeated. Calvariae were then minced into fine pieces with sterile scissors and incubated for 1 hour (h) at 37 °C and 5% CO^2^ with digestion medium II (alpha MEM, 0.8% Trypsin 0.2 mg/mL collagenase P and antibiotics). During this 1-h incubation, calvariae pieces were shaken vigorously for 20 s every 5 to 10 min. Calvariae were next cultured overnight at 37 °C and 5% CO^2^ with alpha MEM containing 20% Fetal Bovine Serum (HyClone, #SH3007103) and antibiotics (Penicillin–Streptomycin-Glutamine [Thermo Fisher, #10378016]). To promote cell dissociation, cells were pipetted up and down. Cells were next allowed to attach for 6 h. The attached cells were washed twice with PBS and cultured for 48 h, at which time cells were transferred to 10-cm dishes to maintain, use, or cryopreserve once confluent.

Calvarial cells were cultured in alpha MEM containing 10% Fetal Bovine Serum and 1% antibiotics (Penicillin–Streptomycin–Glutamine). For inhibition of macroautophagy, cells were cultured with 100 nM Bafilomycin A1 (Fisher Scientific, #5084090001) or vehicle (dimethyl sulfoxide [DMSO]) for 4 h. For inhibition of proteasomal degradation, cells were cultured with 50 μM MG123 (Cell Signaling Technology, #2194) or vehicle (DMSO) for 6 h. Cells were next washed, and protein was extracted as explained below.

### Immunoblots

Protein was extracted from the calvarial cultures using RIPA Buffer (Fisher Scientific, # PI89901) according to the manufacturer’s instructions. Proteins were then resolved in 4–20% or 4–15% Mini-PROTEAN TGX gels (BIORAD, Cat# 4561093 and Cat# 4561083). Proteins were transferred onto TransblotTurbo Midi-size nitrocellulose membranes (0.2 μm pore size, BIORAD, Cat# 1704271). The membranes were blocked for 30 min with LICOR Blocking Buffer-PBS (LICOR, Cat# 4561083) and incubated overnight with primary antibodies and rocking at 4 °C. The primary antibodies used were LAMP2A (Novus Biologicals, # NBP267298), LC3 (Cell Signaling Technology, # 12741), β-Actin (Millipore Sigma, A5316), Ubiquitin (Cell Signaling Technology, #43124), K48-Ubiquitin (Cell Signaling Technology, #8081). After overnight incubation, membranes were washed three times with PBS and incubated for 45 min with appropriate secondary antibodies conjugated with IRDye 680 or IRDye 800 dyes (LI-COR). After washing with PBS, membranes were dried in the dark, scanned, and analyzed with an Odyssey IR imaging system (LI-COR) and Image Studio Software.

### Strength measurements

Following microCT scanning, the femurs and L4s were subjected to biomechanical analysis to obtain strength measurements. The femurs were subjected to 3-point bending on an Instron model 5542 with a ramp rate of 1 mm/min and an 8.1 mm support span. The analysis was run using Bluehill2 software ver. 2.35. The vertebrae were subjected to a vertebral compression test on the Instron 5542 with a ramp of 0.5 mm/min ^50^.

### Statistical analysis

All values are reported as mean ± standard deviation (STDEV). Differences between the two genotypes (WT vs. L2ACgKO) were evaluated using Graph Pad Prism 7.05 software (GraphPad Software, Inc., La Jolla, CA, USA). Unless otherwise indicated, data were analyzed by an unpaired *t*-test with Welch’s correction (does not assume equal variation).

## Supplementary Information

Supplementary Figures.
