# Supplementary material for: Loss of chaperone-mediated autophagy is associated with low vertebral cancellous bone mass
Source: Sci Rep. 2022 Feb 24;12:3134. doi: 10.1038/s41598-022-07157-9 (PMC8873216; doi:10.1038/s41598-022-07157-9)
Supplement: Supplementary file 1 — Supplementary Figures. [file 41598_2022_7157_MOESM1_ESM.pdf]

Loss of chaperone-mediated autophagy is associated with low vertebral cancellous bone mass

Nisreen Akel[1], Ryan S. MacLeod[2,3], Stuart B. Berryhill[5], Dominique J. Laster[1], Milena Dimori[1], Julie A. Crawford[5], Qiang Fu[2, 4] and Melda Onal[1]*

[1]Department of Physiology and Cell Biology, University of Arkansas for Medical Sciences, Little Rock, AR, USA

[2]Center for Musculoskeletal Disease Research, University of Arkansas for Medical Sciences, Little Rock, AR, USA

[3]Division of Endocrinology, University of Arkansas for Medical Sciences, Little Rock, AR, USA

[4]Genetic Models Core Facility, University of Arkansas for Medical Sciences, Little Rock, AR, USA

[5]Bone Histology and Imaging Core, University of Arkansas for Medical Sciences, Little Rock, AR, USA

* Corresponding Author

E-mail: monal@uams.edu (MO)

SUPPLEMENTARY FIGURE LEGENDS

Supplementary Figure 1. Expression of *Lamp2* isoforms in different tissues. Femur shafts, lumbar vertebrae 5 (L5), kidneys, and livers were isolated from the 18-week-old male (**a-b**) and female (**c-f**) wild type (WT) and LAMP2AC global knockout (L2ACgKO) mice. **a-f**, mRNA levels of *Lamp2* isoforms in these tissues were measured by quantitative real-time PCR (qRT-PCR) and normalized to β-actin. Bars indicate mean ± STDEV, n= 6-10 mice per genotype. mRNA values of each isoform from WT and L2ACgKO groups were compared by Welch's *t*-test.

Supplementary Figure 2. Determining the deletion in L2ACgKO mice. Illustration of Chromosome X (Chr X): 38,401,341 -38,456,463 containing the *Lamp2* gene. Schematic of spliced mRNAs encoding the three *Lamp2* isoforms as designated by Ensembl genome browser (Lamp2 ENSMUSG00000016534). Red arrows indicate the locations that the CRISPR-Cas9 sgRNAs target. Expanded view of the ChrX:38,391,630-38,405,790 region in wild-type mice and the sequencing result of the same region in L2ACgKO mice. Black bars represent DNA, gray boxes indicate exons, and small dashed lines indicate deleted/missing regions. The sequence of this locus in L2ACgKO after CRISPR-Cas9 genome editing is indicated below the illustration.

Supplementary Figure 3. Gene expression analysis of bone formation and resorption marker genes in bones of L2ACgKO male mice. qRT-PCR was used to measure mRNA levels of target genes in lumbar vertebrae 5 (L5) of 5-week-old male Lamp2AC global knock-out (L2ACgKO) mice and their littermate controls (wild-type mice, WT). **a-d**, mRNA levels of osteoblast marker gene *Bglap* encoding Osteocalcin (**a**), osteoclast marker genes C*athepsin K (CtsK)* and *Trap (Acp5)* (**b** and **c**)*, Tnfsf11 encoding* pro-resorptive cytokine RANKL (**d**) and *Tnfrsf11b* encoding the decoy receptor of RANKL (**e**) were measured. Levels of target genes were normalized to the housekeeping gene β-actin. n= 8-12 mice/group. Bars indicate mean ± STDEV. *p* values were calculated and evaluated by Welch's *t*-test; *, p<0.05.

Supplementary Figure 4. Biomechanical analysis of young adult L2ACgKO mice. a-b, Biomechanical analysis was performed on femurs (**a**) and lumbar vertebrae 4 (L4) (**b**) of 18-week-old male L2ACgKO mice and their control littermates. Young's modulus, yield stress, and ultimate stress measurements were obtained from three-point bending test of femurs (**a**) or compression test of L4 (**b**). n= 8 mice/group. Bars indicate mean ± STDEV. *p* values were calculated and evaluated by Welch's *t*-test; *, p<0.05.

Supplementary Figure 5. CMA-deficiency is associated with increased osteoclastogenesis. Bone marrow was isolated from 4-month-old L2ACgKO and WT mice. **a**, For each in vitro osteoclastogenesis assay, bone marrow of 3 mice/genotype were pooled and plated in 6-well plates. **a**, Osteoclast number per well (OcN/well) was quantified in each experiment using 3-6 wells/genotype. Bars indicate mean ± STDEV. *p* values were calculated and evaluated by Welch's *t*-test; *, p<0.05. **b**, Representative images of TRAP-stained osteoclasts and their support cells are presented. In each image, osteoclasts are defined as TRAP-positive cells with three or more nuclei, and are indicated with stars (*****). In all images, white scale bars are 200µm.

Supplementary Figure 6. CMA deficiency increases RANKL expression in calvarial osteoblasts. a-c, *Lamp2a* was knocked down in osteoblastic UAMS-32 cells and plated into 6 well plates for gene expression analysis. **d-f,** Wild type calviarial osteoblasts were plated into 6 well plates and each well was stably transfected with scrambled shRNA (shRNA Con) or shRNA targeting LAMP2A (shRNA L2A). **a-f,**

qRT-PCR analysis of three *Lamp2* isoforms and *Tnfsfs11 (Rankl)* in osteoblastic UAMS-32 cells (**a-c**) or calvarial osteoblasts (**d-e**) that stably express scrambled shRNA (shRNA Con) or shRNA targeting LAMP2A (shRNA L2A). mRNA levels of all transcripts were normalized to the housekeeping gene β-actin. n= 3 wells/group. Bars indicate mean $\pm$ STDEV. *p* values were calculated and evaluated by student's *t*-test; *, p<0.05.

Supplementary Figure 7. Macroautophagy and proteasomal degradation levels in L2ACgKO calvarial osteoblasts. Calvarial osteoblasts were isolated from Lamp2AC global knock-out (L2ACgKO) mice and their control littermates (wild-type mice, WT). **a**, Original images of western blot analysis as shown in Fig. 7. **b**, Calvarial osteoblasts isolated from one mouse per genotype were plated in 6 well plates and cultured with vehicle (DMSO) or 50µM of proteasome inhibitor MG132 for 6 hours, and western blot analysis was performed. For this purpose, one membrane was incubated with antibodies against actin (red) and ubiquitin (green). Another membrane was incubated with antibodies against actin (red) and K48-linked ubiquitination (green). **c**, Calvairal osteoblasts isolated from a second set of WT and L2ACgKO mice were cultured with vehicle (DMSO) or 100µM Bafilomycin A (BafA) for 4 hours. LAMP2A, LC3, and p62 protein levels were determined by western blot analysis. LC3-II and p62 levels were quantified and normalized to actin levels. Original images of western blot membranes of this experiment are shown in **d**. For western blot analysis of macroautophagy in both experiments (**a** and **d**), one membrane was incubated with antibodies against actin (red) and LAMP2A (green). Another membrane containing the WT and L2ACgKO samples was cut into two at 25kDa. The top part of this membrane was incubated with antibodies against actin (red) and p62 (green). The bottom part of the same membrane was incubated with antibodies targeting LC3 (green). All membranes were incubated with appropriate secondary antibodies conjugated with IRDye 680 or IRDye 800 dyes (LI-COR), scanned, and analyzed as described in the method section.

S. Figure 1

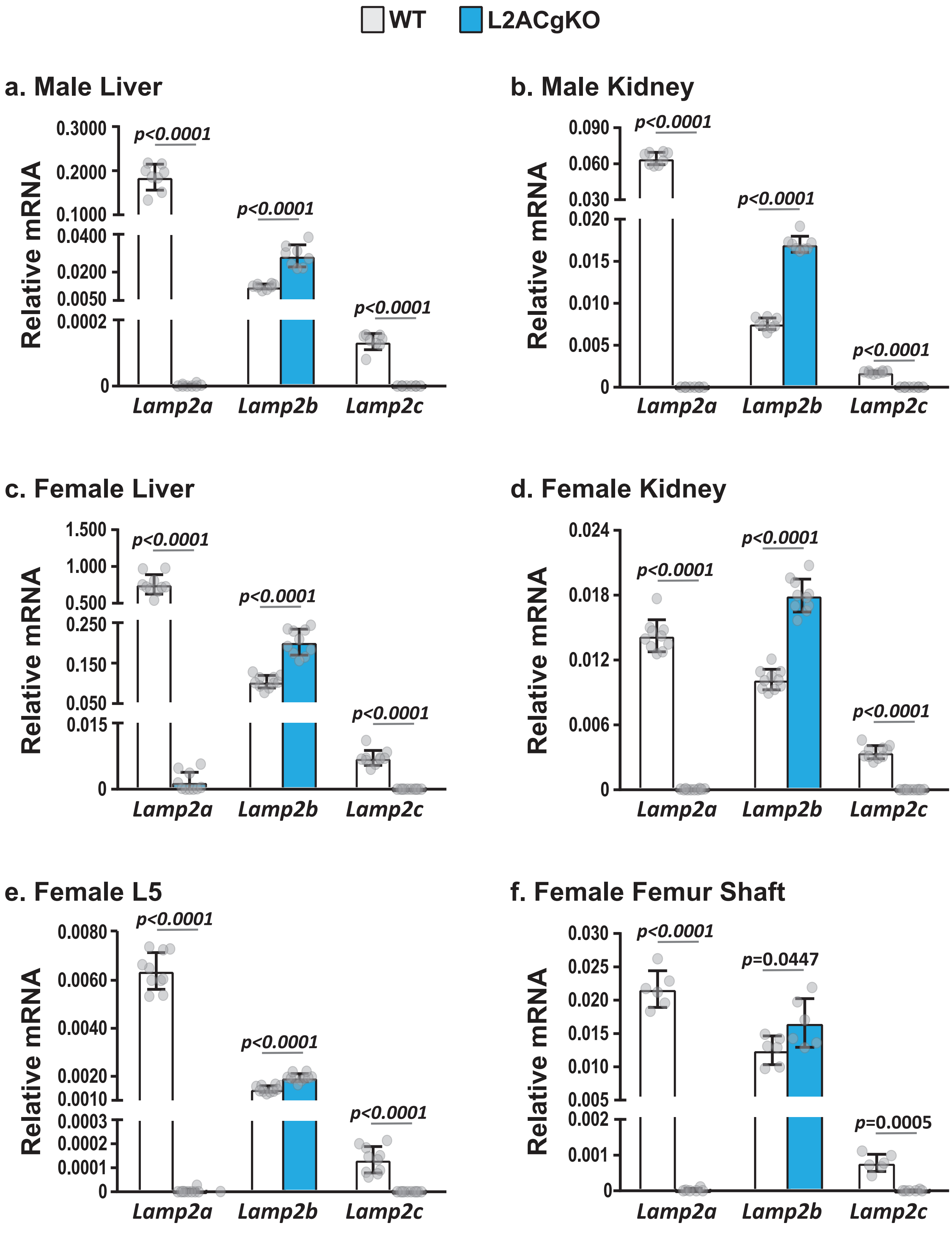

WT
L2ACgKO
a. Male Liver
b. Male Kidney
c. Female Liver
d. Female Kidney
e. Female L5
f. Female Femur Shaft
Relative mRNA
p<0.0001
p=0.0447
p=0.0005
Lamp2a
Lamp2b
Lamp2c

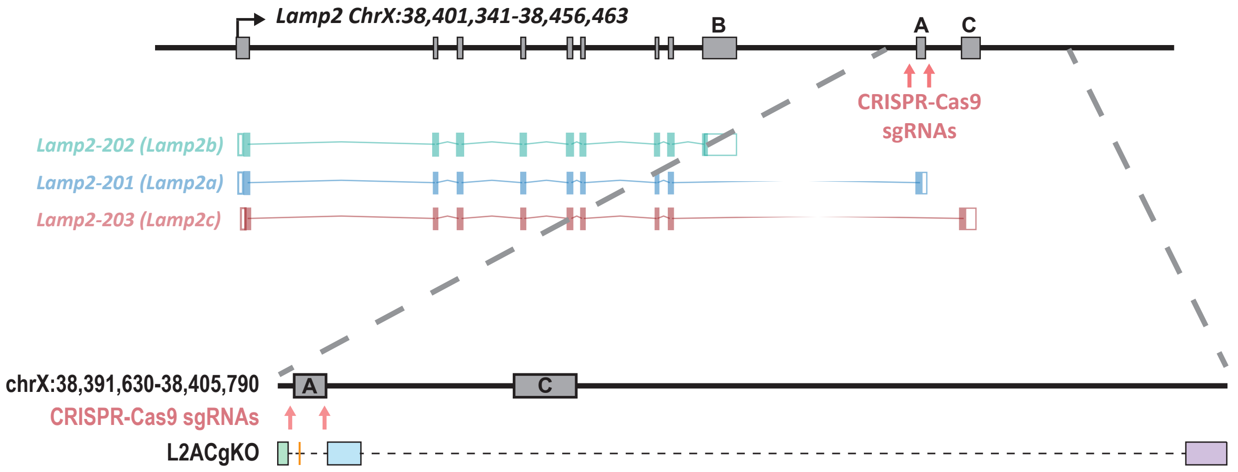

Sequence of L2ACgKO:

GCATTGAGCTCTGTGTCTGCTTTATATACGTGTTTATTTGGTGAGTTAGTACTCTCTAATATCGAGGATTCAATGTCA
TAGTTGGCTCTGGAAGCTCCAGGCTGATCAAGGGAAAGCAAATTTCTGTAAGAAGCCTGGGAGTGTGTGCTCACTATA
CACATTTTCCCCCCAATGACTGCTTTTTATGAAGGGGAACTGCATAGAAGTACTTGTTTTTTGTTTTTGCTTGAAAAA
AAAAACCTTTGGTGCAACATCTCATGAAATATCATATCACTACTGATAATGAATGCCAATGACTTGCCATTTTGATGT
AAAAATCTGTATGCAGGGGTCAAGTGTTCTGTTTCAATTTGTAACAGCTGTCCTAATTTAAACTCACCACTACTAGCT
TTTGCATATACTTAAAATACCTATCAGATATTCACAGACTTGGTTTTTGGAGGGATGAAGTAGAGAGTCCGTAGGGCC
ATCCACAGTTTGTTTTTGTTTTTTGTTTTTTAAATAAGAACTGAAGAGGAACCAGAGAGTGTTTGTTTGTTTGGTTTT
TTGAAACAGGGTTTCTCTGTGTAGCCCTTGTCCTGGAAGTCACTCTTTAGACCAGGCTGGCCTTGAACTCACAGAGAT
CCACCTGCTTCTGCCTCCTGAATGCTGAAATTGAGGTGTGTGCTATCGCCACCCAGCTAAGTATTACATTTTTAAAGG
TTATGACTTGATGTCTTTTGAGTTCAAGTTAAATATGAAATTGATTCTCAAACTTGTGATTTTTTTACATAGGTTAAA
AACAGGCTCAGAAAGTTATAACAGAGCTCAGACTGGCTTCTTAGGTTGTTTTGTTTTGTTGTTATTTTGAGACAGGGT
TTCTCTGTGTAACTTTGGCTATCCTGGAACTTGATCTGTAGACCAGGCTGGCCTCAAAGTTAGAGATTCACCTGCCTC
TTCCTCATAAGTGTTGGGATTAAAGTCATGCTCCACCACTGCCTGGCTAACATGTAGTGTGTGTGTGTGTGTGTGTGT
GTGTGTGTGTGTGTGTGAATTTATAGGGTTATTCTCTTCAATCAGTAGGGAAAGAGTGGAACATTAAAAATAATGCTT
AACTATTGAAACAGTAGTTTACTTATTTTTAGACCCCTGACTTCACAACATTAAAAAAATGCAAGTATGCTTAAAGAG
GTTAAAATTTAAACTACAGAAGATCCAGAATTGAACATAGATAGCTACAGTAGCACAGTAGTGGGGATGTTGTGGTAG
AAACACTTGCTTGGCATGCACAAGGCTGTGAAATAATCCACCCCCCACGAGCACATACACACGAGTGAAAAATGATAG
TGGAAAAGAAGATATTTCAATATTTAAAAATGTACTATTTCTGAGCANNTAAGTGACACCAGACACAAAGTTNAACAA

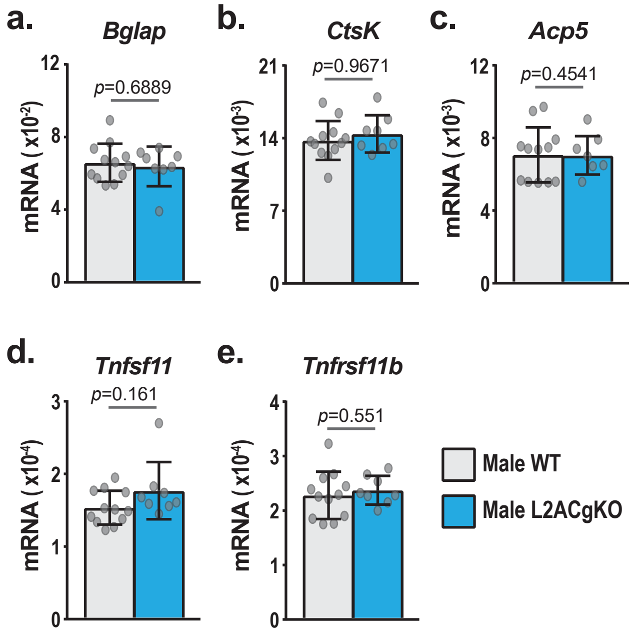
a.
Bglap
p=0.6889
mRNA (x10-2)
0
4
8
12
b.
CtsK
p=0.9671
mRNA (x10-3)
0
7
14
21
c.
Acp5
p=0.4541
mRNA (x10-3)
0
4
8
12
d.
Tnfsf11
p=0.161
mRNA (x10-4)
0
1
2
3
e.
Tnfrsf11b
p=0.551
mRNA (x10-4)
0
1
2
3
4
Male WT
Male L2ACgKO

S.Figure 4

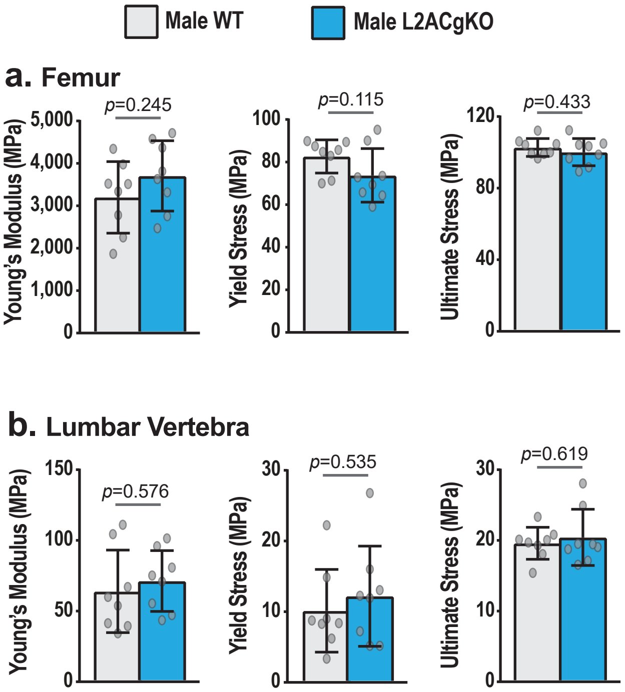

Male WT
Male L2ACgKO
a. Femur
Young's Modulus (MPa)
p=0.245
Yield Stress (MPa)
p=0.115
Ultimate Stress (MPa)
p=0.433
b. Lumbar Vertebra
Young's Modulus (MPa)
p=0.576
Yield Stress (MPa)
p=0.535
Ultimate Stress (MPa)
p=0.619

S.Figure 5

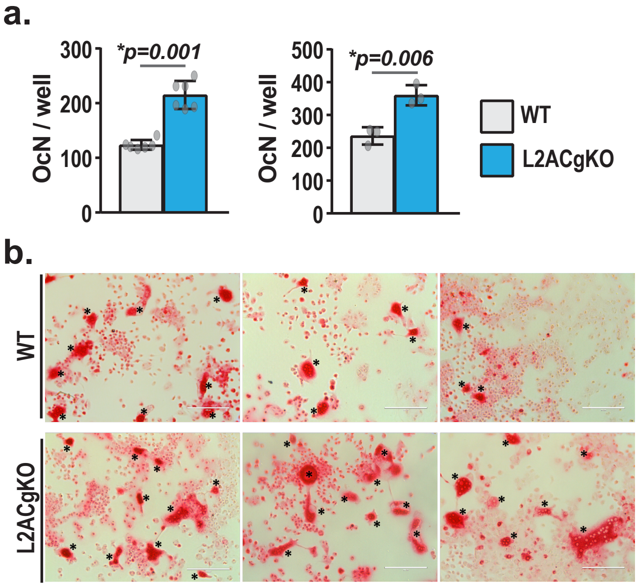
a.
*p=0.001
300
200
100
0
OcN / well
*p=0.006
500
400
300
200
100
0
OcN / well
WT
L2ACgKO
b.
WT
L2ACgKO

S.Figure 6

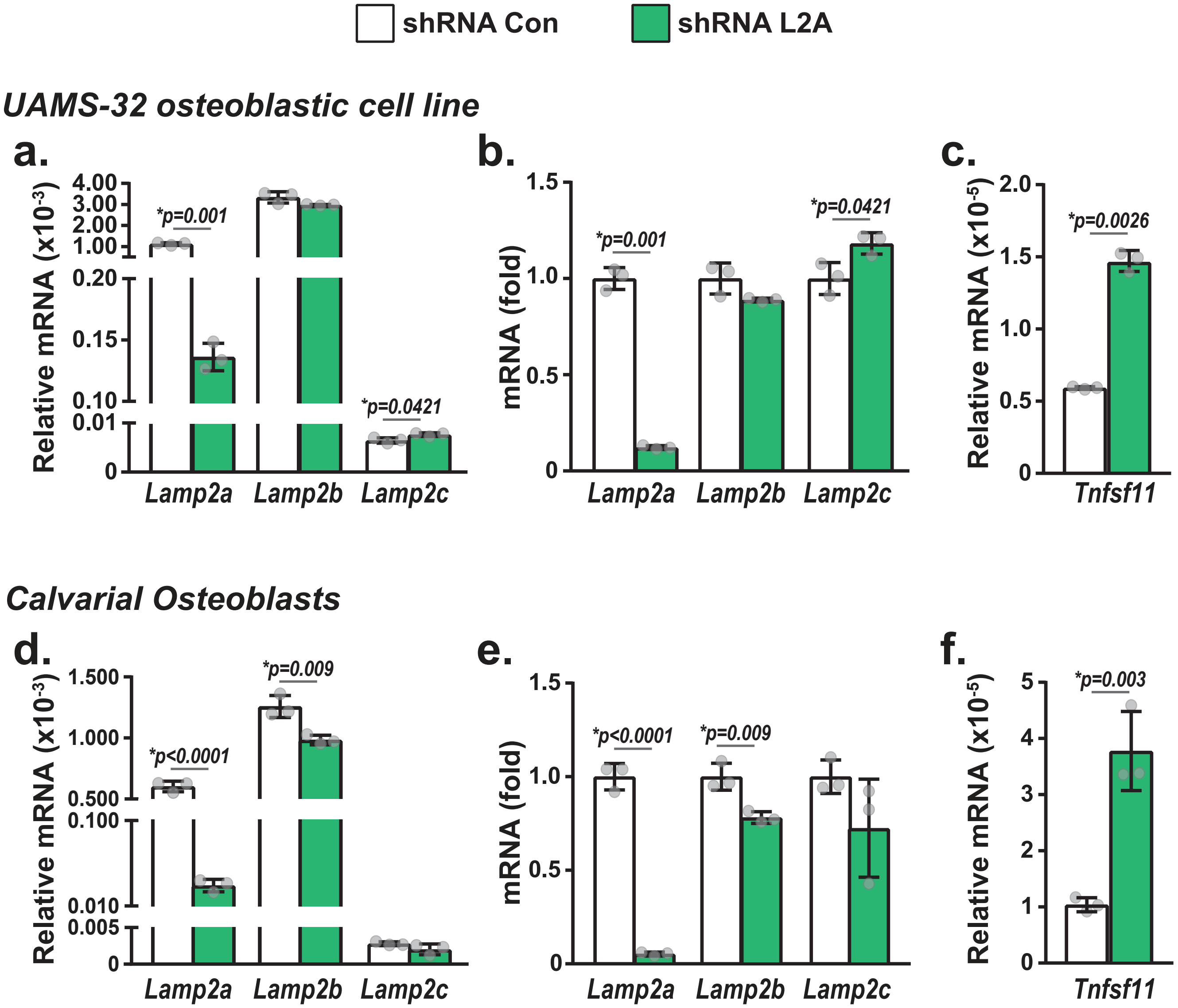
shRNA Con
shRNA L2A
UAMS-32 osteoblastic cell line
a.
Relative mRNA (x10-3)
*p=0.001
*p=0.0421
Lamp2a
Lamp2b
Lamp2c
b.
mRNA (fold)
*p=0.001
*p=0.0421
Lamp2a
Lamp2b
Lamp2c
c.
Relative mRNA (x10-5)
*p=0.0026
Tnfsf11
Calvarial Osteoblasts
d.
Relative mRNA (x10-3)
*p<0.0001
*p=0.009
Lamp2a
Lamp2b
Lamp2c
e.
mRNA (fold)
*p<0.0001
*p=0.009
Lamp2a
Lamp2b
Lamp2c
f.
Relative mRNA (x10-5)
*p=0.003
Tnfsf11

S. Figure 7

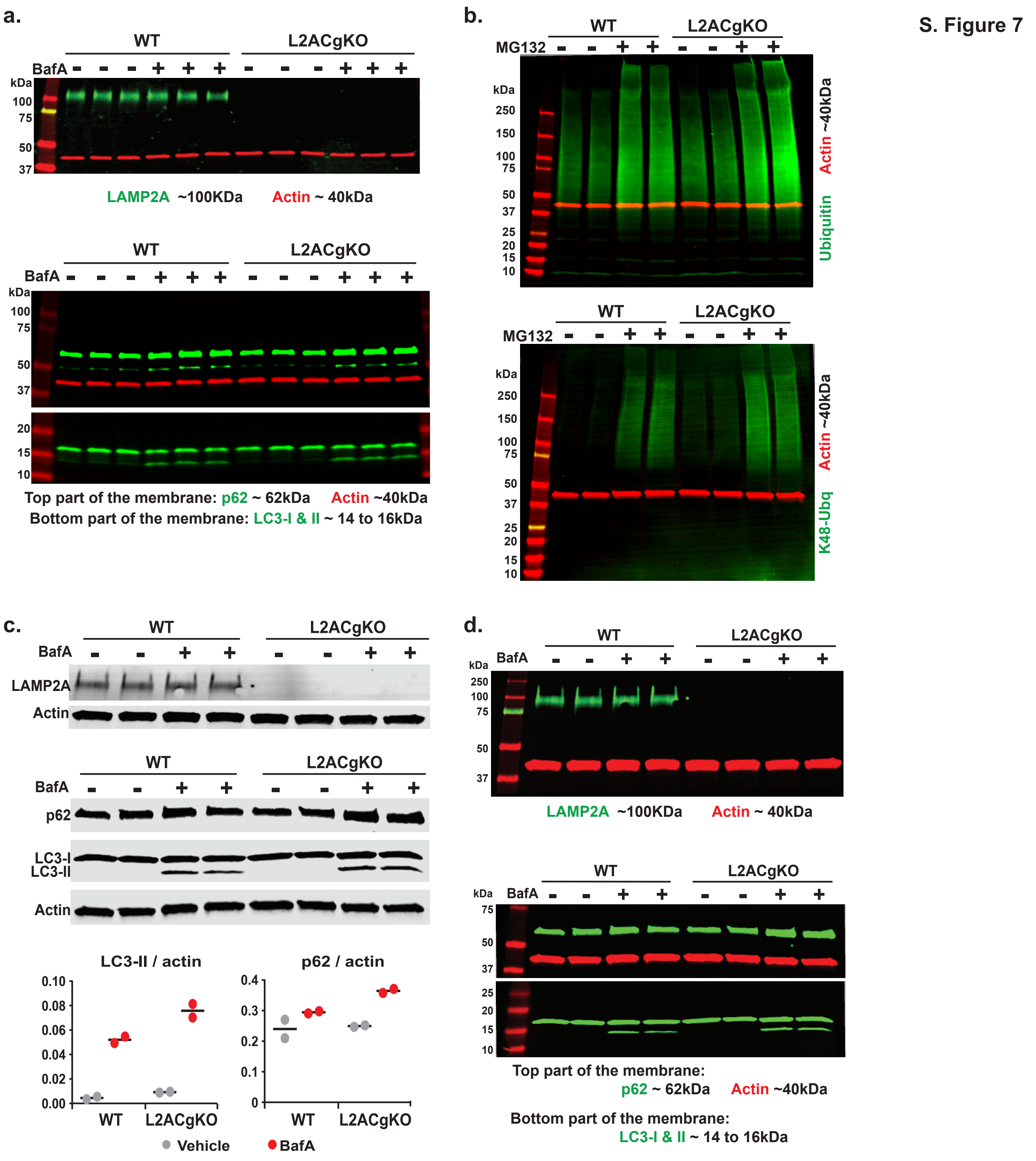
a.
WT
L2ACgKO
BafA - - - + + + - - - + + +
kDa
100
75
50
37
LAMP2A ~100KDa
Actin ~ 40kDa
WT
L2ACgKO
BafA - - - + + + - - - + + +
kDa
100
75
50
37
20
15
10
Top part of the membrane: p62 ~ 62kDa Actin ~40kDa
Bottom part of the membrane: LC3-I & II ~ 14 to 16kDa
b.
WT
L2ACgKO
MG132 - - + + - - + +
kDa
250
150
100
75
50
37
25
20
15
10
Actin ~40kDa
Ubiquitin
WT
L2ACgKO
MG132 - - + + - - + +
kDa
250
150
100
75
50
37
25
20
15
10
Actin ~40kDa
K48-Ubq
c.
WT
L2ACgKO
BafA - - + + - - + +
LAMP2A
Actin
WT
L2ACgKO
BafA - - + + - - + +
p62
LC3-I
LC3-II
Actin
LC3-II / actin
0.10
0.08
0.06
0.04
0.02
0.00
WT
L2ACgKO
p62 / actin
0.4
0.3
0.2
0.1
0
WT
L2ACgKO
Vehicle
BafA
d.
WT
L2ACgKO
kDa
BafA - - + + - - + +
250
100
75
50
37
LAMP2A ~100KDa
Actin ~ 40kDa
WT
L2ACgKO
kDa
BafA - - + + - - + +
75
50
37
25
20
15
10
Top part of the membrane:
p62 ~ 62kDa Actin ~40kDa
Bottom part of the membrane:
LC3-I & II ~ 14 to 16kDa
